# Pulmonary Paragonimiasis Presenting as Pneumothorax, Hemoptysis, and Severe Eosinophilia: A Case Report

**DOI:** 10.1155/crdi/1991391

**Published:** 2026-06-21

**Authors:** Ashok Basnet, Mukti Nath Sankhi, Chiranjibi Pant, Rohit Vashisht, Alina Singh, Brikshya Gurung, Bishal Kunwor

**Affiliations:** ^1^ Department of Pulmonary Medicine, Nepalese Army Institute of Health Sciences College of Medicine, Kathmandu, Bagmati, Nepal; ^2^ Department of Internal Medicine, Nepalese Army Institute of Health Sciences College of Medicine, Kathmandu, Bagmati, Nepal; ^3^ Department of Internal Medicine, Army Hospital Research and Referral, New Delhi, Delhi, India; ^4^ Department of Microbiology, Nepalese Army Institute of Health Sciences College of Medicine, Kathmandu, Bagmati, Nepal; ^5^ Department of Radiodiagnosis, Nepalese Army Institute of Health Sciences College of Medicine, Kathmandu, Bagmati, Nepal

## Abstract

Paragonimiasis is a foodborne zoonosis transmitted mostly by the consumption of inadequately cooked crabs and crayfish harboring *Paragonimus* metacercariae. We present the case of a 38‐year‐old male with a history of crab ingestion who developed severe eosinophilia, transaminitis, and serositis. Initial presentation included fever, epigastric pain, weight loss, and pleural effusion. He was managed empirically with albendazole and corticosteroids along with other supportive measures. Two months later, he re‐presented with hemoptysis and hypoxemia. HRCT showed multiple cavitary pulmonary lesions, pleural nodules, and left‐sided pneumothorax. Sputum wet mount demonstrated *Paragonimus* species ova, confirming pulmonary paragonimiasis. The patient was managed with intercostal tube drainage, tranexamic acid nebulization, and praziquantel with clinical improvement. This case highlights the importance of considering paragonimiasis in patients, even with pneumothorax, when the patient has hemoptysis and eosinophilia in the background of ingestion of raw or undercooked crustaceans in endemic regions.

**Key Clinical Message**
 Paragonimiasis should be considered by all physicians when a patient presents with cough, hemoptysis, and even pleural effusion or pneumothorax, particularly when there is a history of ingestion of raw or undercooked freshwater crabs or crayfish. This condition can closely mimic pulmonary tuberculosis, leading to misdiagnosis in endemic settings. Early recognition and appropriate diagnostic evaluation are crucial to ensure timely treatment and avoid unnecessary antitubercular therapy.

## 1. Introduction

Paragonimiasis is a tropical disease caused by trematodes of the genus *Paragonimus*, with *Paragonimus westermani* being the most common human pathogen. Globally, paragonimiasis is endemic primarily in East and Southeast Asia; however, it has also been reported in parts of West and Central Africa and in Central and South America. Eight species can infect humans. Three human‐infecting species are distributed in Asia, where *P. westermani* has the broadest distribution (East Asia, the Far East, Southeast Asia, India, Sri Lanka, probably Nepal, and New Guinea) not only in Asia but also in the world. *P. skrjabini* is distributed mainly in China and Japan. *P. heterotremus* is limited to the Indochina Peninsula and southwestern border regions of China and northeastern India. There are two human‐infecting *Paragonimus* species distributed in the Americas: *P. kellicotti* and *P. mexicanus*. The two species are entirely geographically separated: *P. kellicotti* is distributed in North America, whereas *P. mexicanus* is distributed in Latin America. Three human‐infecting species have been reported in Africa: *P. africanus*, *P. uterobilateralis,* and *P. gondwanensis*. The three species are mostly distributed in West and Central Africa [1]. Few cases have been reported from Nepal [2]. Transmission of the disease occurs through ingestion of raw or undercooked freshwater crabs or crayfish containing metacercariae [3]. Clinical manifestations can mimic tuberculosis, bacterial pneumonia, or malignancy. Severe eosinophilia, pleural effusion, and cavitary lung lesions are common but underrecognized. Pneumothorax and hemoptysis are rare but serious complications. This case illustrates diagnostic challenges and emphasizes the importance of a detailed dietary history and prompt parasitological investigation.

## 2. Case Presentation

### 2.1. Case History and Examination

A 38‐year‐old male presented to the emergency department of a tertiary care center with a 2‐week history of intermittent fever, reaching a maximum of 101 F. The fever was more pronounced in the evenings and was accompanied by chills. In addition, he noted a loss of appetite, significant weight loss of about 5 kg, intermittent shortness of breath, and a dry cough of 1 month′s duration. The onset of symptoms followed ingestion of freshwater crab. On admission, the patient was afebrile with a blood pressure of 110/60 mmHg, heart rate of 76 beats/min, respiratory rate of 22 breaths/min, and oxygen saturation of 98% on room air. Systemic examination revealed no significant findings.

### 2.2. Diagnosis, Investigation, and Treatment

Laboratory evaluation revealed marked eosinophilia (total leukocyte count 23,560/μL [reference: 4000–11000] with 60.96% eosinophils [reference: 1%–6%]) and elevated liver enzymes: Aspartate aminotransferase (AST): 107 U/l (reference: 13–40), alanine aminotransferase (ALT): 277 U/l (reference: 13–40), and alkaline phosphatase 286 U/L (reference: 40–120), suggestive of transaminitis. Chest X‐ray showed left‐sided pleural effusion (Figure 1). Stool examination was performed as part of the eosinophilia workup, where the test did not reveal any ova cyst of parasites. Sputum examination could not be performed as the patient did not have expectoration.

**FIGURE 1:**
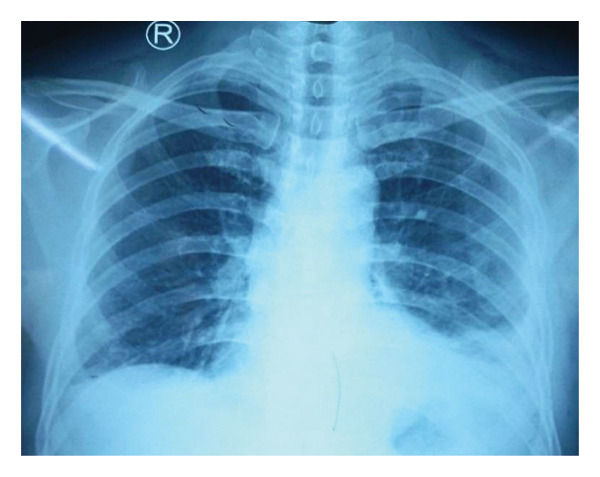
Chest radiograph posteroanterior view showing left‐sided pleural effusion.

He was managed as a case of severe eosinophilia, associated with transaminitis and serositis likely due to parasitic infection on the background of crab ingestion. Treatment included high‐dose corticosteroids (tab prednisolone) for 6 weeks in tapering doses and empirical anthelminthic (oral albendazole) for 3 days along with supportive therapy. He improved clinically and was discharged with a tapering course of steroids and regular follow‐up advice. However, he was lost to follow‐up.

## 3. Subsequent Presentation

### 3.1. Case History and Examination

Two months later, the patient presented again with hemoptysis for 2 days, described as approximately one teaspoon of blood per episode, associated with left‐sided pleuritic chest pain and shortness of breath. On examination, his blood pressure was 110/70 mmHg, pulse 110 beats/min, respiratory rate 26 breaths/min, temperature 98 F, and oxygen saturation 90% on room air. Breath sounds were absent over the left hemithorax.

### 3.2. Diagnosis, Investigation, and Treatment

A chest X‐ray revealed a large left‐sided pneumothorax (Figure 2). HRCT of the chest demonstrated multiple small cavitary lesions and pleural‐based nodules along with left‐sided pneumothorax (Figure 3).

**FIGURE 2:**
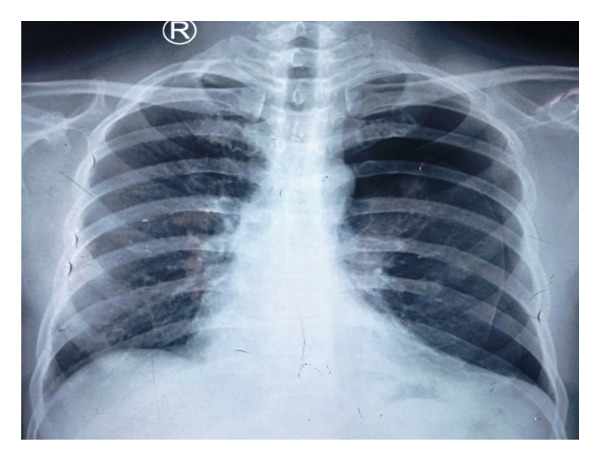
Chest radiograph posteroanterior view showing left‐sided pneumothorax.

**FIGURE 3:**
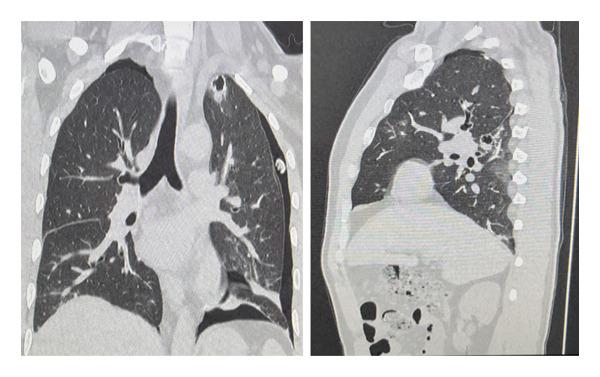
High resolution computed tomography image showing left‐sided pneumothorax, multiple small cavitary lesions, and pleural‐based nodules.

Sputum analysis showed > 25 pus cells per low‐power field and a mixed growth of commensal bacteria. Evaluation for tuberculosis (sputum acid‐fast bacilli smears and GeneXpert) was negative. However, sputum wet mount and Ziehl–Neelsen stain examination revealed characteristic operculated egg of *Paragonimus* species, confirming the diagnosis of pulmonary paragonimiasis (Figures 4 and 5).

**FIGURE 4:**
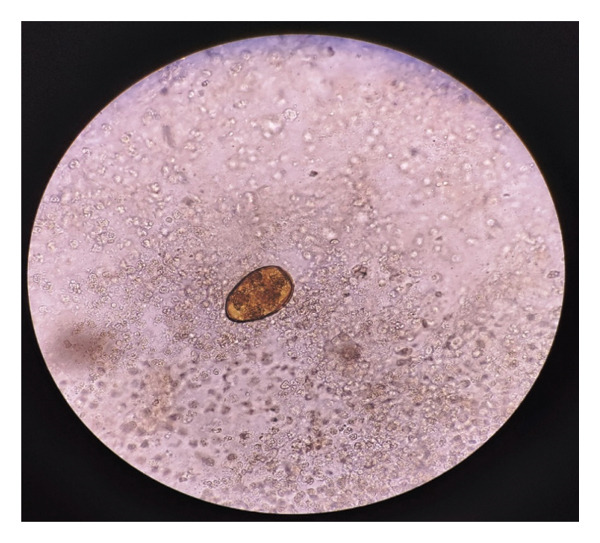
Wet mount preparation of a sputum sample showing *Paragonimus* species egg.

**FIGURE 5:**
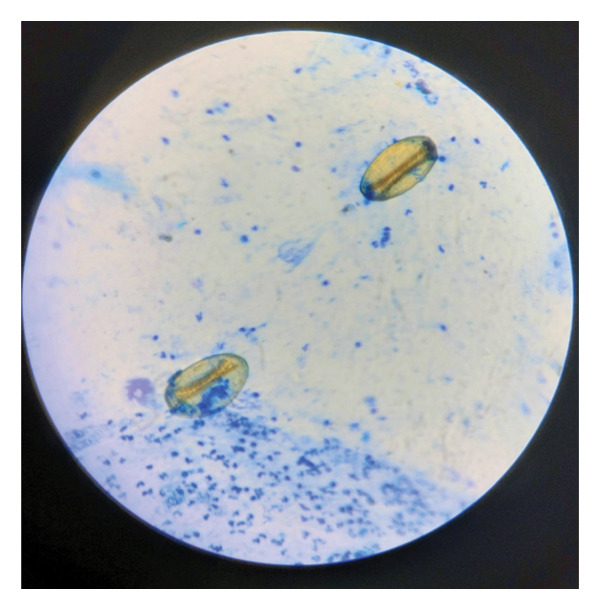
Ziehl–Neelsen stain of a sputum sample showing yellow–brown, ovoid, thick‐shelled, with a flattened operculum at one end, consistent with *Paragonimus* species.

He was managed with intercostal tube drainage for pneumothorax and tranexamic acid nebulization. Specific antiparasitic therapy was started with praziquantel at a dose of 25 mg/kg per dose, three times daily for 3 days. Oral corticosteroid was continued in a tapering dose and gradually stopped.

### 3.3. Outcome and Follow‐Up

The patient showed gradual improvement in respiratory symptoms and resolution of pneumothorax. On follow‐up assessment, the patient is asymptomatic with radiological improvement. Follow‐up sputum examination did not reveal the parasite. The individual had a resolution of the cough subsequently.

## 4. Discussion

*Paragonimus* species has a complex life cycle involving two intermediate hosts—a freshwater snail and a crustacean such as a crab or crayfish—and a definitive mammalian host [1]. Humans acquire infection by ingesting raw or undercooked crustaceans harboring metacercariae, the infective stage [1]. Once ingested, the metacercariae excyst in the duodenum, penetrate the intestinal wall, and migrate through the peritoneal cavity and diaphragm to reach the lungs, where they mature into adult flukes and begin egg production within 2–3 months [4]. Rarely, larvae may reach ectopic sites such as the brain, muscles, or subcutaneous tissue, causing extrapulmonary manifestations [4]. This migratory behavior underlies the diverse clinical presentations observed in paragonimiasis. Our case has a history of crab ingestion.

The clinical manifestations of *Paragonimus* species infection result from larval migration and host immune response. Migration to the lungs causes tissue injury, inflammation, and hemorrhage, leading to cough, hemoptysis, pleuritic pain, and, in severe cases, pneumothorax due to rupture of subpleural cysts or cavitary lesions [5, 6]. Egg deposition induces pleural effusions and pulmonary nodules. Ectopic migration to organs such as the liver or brain may cause transaminitis or neurological symptoms, while marked eosinophilia reflects the systemic immune response to the parasite [7].

Diagnosis of *Paragonimus* species relies on clinical suspicion, exposure history, eosinophilia, and imaging showing pleural effusion, pulmonary nodules, cavitary lesions, or pneumothorax. Definitive confirmation is achieved by detecting characteristic ova in sputum, stool, or bronchoalveolar lavage [8]. Demonstration of ova in expectorated sputum is specific; however, the sensitivity of this test is low (28%–38%). Stool examination has low sensitivity, and the ova are not usually found in pleural fluid [9]. Serological testing for anti‐*Paragonimus* antibody by enzyme‐linked immunosorbent assay (ELISA) has a sensitivity of 100% and a specificity of 91%–100% [10]. Praziquantel or triclabendazole can be used for the treatment of paragonimiasis. Praziquantel at the dose of 25 mg/kg is given orally three times daily for 2‐3 days, whereas triclabendazole is used as two doses of 10 mg/kg given 12 h apart [11, 12]. Our patient received empirical treatment with albendazole during the initial visit; however, the therapy was not completely effective, as the patient later presented with progression despite initial improvement. This might be due to reduced susceptibility or possible resistance of the helminth to the drug. However, it requires further study for confirmation. Supportive care addresses hemoptysis, effusions, or pneumothorax and relieves symptoms with corticosteroids or antihistamines as needed. Our patient showed rapid improvement after receiving praziquantel therapy. In our case, we used nebulized tranexamic acid for the management of hemoptysis. A meta‐analysis demonstrated that patients receiving nebulized tranexamic acid were 3.85 times more likely to achieve hemoptysis cessation than those receiving alternative treatments. Moreover, patients receiving nebulized tranexamic acid required fewer (43%) pulmonary interventional procedures than those receiving other treatments [13].

Paragonimiasis frequently mimics tuberculosis or bacterial pneumonia. In places where TB prevalence is high, patients are often treated empirically for TB before parasitic infection is considered [14]. A detailed dietary history and eosinophil count are critical diagnostic clues.

## 5. Conclusion

Paragonimiasis is endemic in many parts of Nepal. There have been reports of paragonimiasis presenting with pleural effusion, hemoptysis, and a persistent cough from South Asia. However, cases with documented pneumothorax and marked transaminitis remain rare. Our case highlights the value of early parasitological testing before the patient develops complications. Every physician must be aware of this tropical disease to avoid misdiagnosis and treatment.

## Author Contributions

All authors contributed to the manuscript. Ashok Basnet, Alina Singh, and Brikshya Gurung contributed to data collection. The first draft of the manuscript was written by Mukti Nath Sankhi, Ashok Basnet, and Bishal Kunwor, revised by Chiranjibi Pant and Rohit Vashisht.

## Funding

No funding was obtained for this study.

## Disclosure

All authors have read and approved the final manuscript.

## Consent

Written informed consent was obtained for the publication of this manuscript and accompanying images using the consent form provided by the Journal.

## Conflicts of Interest

The authors declare no conflicts of interest.

## Data Availability

The data that support the findings of this case report are available from the corresponding author upon reasonable request.
